# Culture Independent Genomic Comparisons Reveal Environmental Adaptations for Altiarchaeales

**DOI:** 10.3389/fmicb.2016.01221

**Published:** 2016-08-05

**Authors:** Jordan T. Bird, Brett J. Baker, Alexander J. Probst, Mircea Podar, Karen G. Lloyd

**Affiliations:** ^1^Department of Microbiology, University of Tennessee at Knoxville, KnoxvilleTN, USA; ^2^Department of Marine Science, University of Texas at Austin, Marine Science Institute, Port AransasTX, USA; ^3^Department of Earth and Planetary Science, University of California at Berkeley, BerkeleyCA, USA; ^4^Biosciences Division, Oak Ridge National Laboratory, Oak RidgeTN, USA

**Keywords:** single cell genomics, uncultured archaea, comparative genomics, marine sediment, autotrophy, metagenomics, ecophysiology

## Abstract

The recently proposed candidatus order Altiarchaeales remains an uncultured archaeal lineage composed of genetically diverse, globally widespread organisms frequently observed in anoxic subsurface environments. In spite of 15 years of studies on the psychrophilic biofilm-producing *Candidatus* Altiarchaeum hamiconexum and its close relatives, very little is known about the phylogenetic and functional diversity of the widespread free-living marine members of this taxon. From methanogenic sediments in the White Oak River Estuary, NC, USA, we sequenced a single cell amplified genome (SAG), WOR_SM1_SCG, and used it to identify and refine two high-quality genomes from metagenomes, WOR_SM1_79 and WOR_SM1_86-2, from the same site. These three genomic reconstructions form a monophyletic group, which also includes three previously published genomes from metagenomes from terrestrial springs and a SAG from Sakinaw Lake in a group previously designated as pMC2A384. A synapomorphic mutation in the Altiarchaeales tRNA synthetase β subunit, *pheT*, caused the protein to be encoded as two subunits at non-adjacent loci. Consistent with the terrestrial spring clades, our estuarine genomes contained a near-complete autotrophic metabolism, H_2_ or CO as potential electron donors, a reductive acetyl-CoA pathway for carbon fixation, and methylotroph-like NADP(H)-dependent dehydrogenase. Phylogenies based on 16S rRNA genes and concatenated conserved proteins identified two distinct sub-clades of Altiarchaeales, Alti-1 populated by organisms from actively flowing springs, and Alti-2 which was more widespread, diverse, and not associated with visible mats. The core Alti-1 genome suggested Alti-1 is adapted for the stream environment with lipopolysaccharide production capacity and extracellular hami structures. The core Alti-2 genome suggested members of this clade are free-living with distinct mechanisms for energy maintenance, motility, osmoregulation, and sulfur redox reactions. These data suggested that the hamus structures found in *Candidatus* Altiarchaeum hamiconexum are not present outside of stream-adapted Altiarchaeales. Homologs to a Na^+^ transporter and membrane bound coenzyme A disulfide reductase that were unique to the brackish sediment Alti-2 genomes, could indicate adaptations to the estuarine, sulfur-rich environment.

## Introduction

**FIGURE 1:**
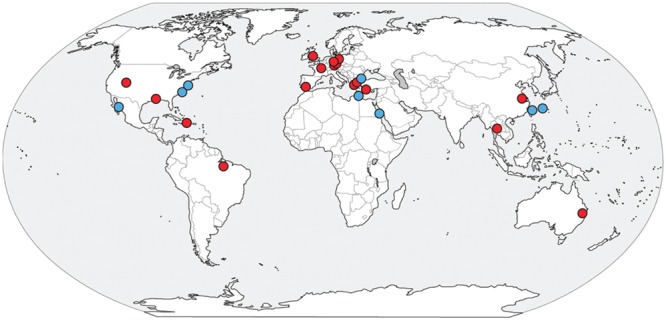
**Global distribution of Altiarchaeales 16S rRNA gene sequences present in the NCBI database**.

An uncultivated group of environmental archaea, originally called the SM1 but recently given the name Altiarchaeales, was first described as a nearly monoclonal biofilm in a cold terrestrial sulfidic spring in Regensburg, Germany (Rudolph et al., 2001; Moissl et al., 2002). The originally described SM1 have unique appendages called hami that work like grappling hooks to maintain their position in the flowing springs (Moissl et al., 2002, 2003, 2005). Altiarchaeales represent one example of biofilm-forming archaea, along with *Sulfolobus* sp. in hot springs (Brock et al., 1972; Zillig et al., 1980; Suzuki et al., 2002), and uncultured ANME archaea in euxinic basins (Michaelis et al., 2002). Their hami structures have no analog in other microbes and might have technological importance due to their intricate nano-sized structure (Perras et al., 2014). Additionally, Altiarchaeales appear to be one of the few examples of archaea with a double cell membrane (Probst et al., 2014; Probst and Moissl-Eichinger, 2015). Furthermore, the Altiarchaeales appear to belong to the phylum Euryarchaeota, which contains most of the industrially and environmentally important archaeal cultures: halophilic phototrophs, sulfate reducers, iron cycling extremophiles, and all cultured methanogens. However, little is known about the functional diversity and evolutionary history of the Altiarchaeales. 16S rRNA gene diversity surveys indicate the Altiarchaeales are a globally distributed group with a wide preference for anoxic environments such as lake sediments, sulfidic aquifers, geothermal springs, deep sea sediments, mud volcanoes, and hydrothermal vents as well as industrial settings and drilled wells (Probst et al., 2014) (**Figure 1**).

Despite the cosmopolitan nature of the Altiarchaeales, these organisms have never been isolated in pure culture, and genomes from metagenomes have only been obtained from terrestrial cold springs. A metagenome from a natural enrichment in a sulfidic spring in Muehlbacher Schwefelquelle, Germany, enabled the assembly of the *Candidatus* Altiarchaeum hamiconexum genome (MSI_SM1), from the visible mats (Probst et al., 2014). MSI_SM1 contained putative genes for the hami as well as conserved evolutionary marker genes that placed it as a new order within the Euryarchaeota (Probst et al., 2014). *Candidatus* Altiarchaeum hamiconexum is naturally enriched in sulfidic springs and hypothesized to play a role in sulfur cycling (Moissl et al., 2002). However, MSI_SM1 contained no genetic evidence for the use of sulfur-containing compounds in respiration. A genome from a less abundant Altiarchaeales (IMC4_SM1) was also reconstructed from the same sample. Another genome reconstructed from subsurface water filtrates from the Crystal Geyser (USA) spring, CG_SM1, was found to be closely related to *Candidatus* Altiarchaeum hamiconexum (Probst et al., 2014). In both cases, these microbes were dominant members of there microbial communities. In depth genomic analysis of MSI_SM1 and CG_SM1 suggested that the Altiarchaeales are autotrophic, utilizing a modified version of the archaeal reductive acetyl-CoA (Wood–Ljungdahl) pathway. Further support for autotrophy comes from the ^13^C-depleted isotope content of the lipid archaeol found at the German site (Probst et al., 2014). MSI_SM1 and CG_SM1 share close evolutionary histories, with >98% identical 16S rRNA genes, and all three genomes from metagenomes were from similar terrestrial cold spring environments. In order to describe the functional diversity and evolutionary radiation of the order Altiarchaeales, it is important to expand the genomic comparison to include distantly related members obtained from different environments.

We obtained genomic reconstructions from brackish sediments in the White Oak River Estuary (WOR), NC, USA. These sediments have a stable redox gradient with microbially mediated sulfate reduction via organic matter oxidation, methane oxidation at sulfate methane transition zone (SMTZ), and then methanogenesis after sulfate is depleted (Martens and Goldhaber, 1978; Kelley et al., 1990; Lloyd et al., 2011). The microbial community is similar to those found in marine sediments worldwide (Lloyd et al., 2011; Kubo et al., 2012).

Similar to the German spring environments and Crystal Geyser, the WOR sediment environment is sulfidic (Rudolph et al., 2004; Probst et al., 2016). Microbial communities deep in the WOR sediment do not experience the active flow regimes of those microbial communities in the German spring and Crystal Geyser. In contrast, the WOR sediments have much more stable flow regimes and redox gradients that shift on longer seasonal timescales (Lloyd et al., 2011). In WOR sediments, microorganisms demonstrate a clear effect on the geochemical environment as vertical profiles often reveal hierarchical zonation based available highest energy electron acceptors (Kelley et al., 1990). Furthermore, deep in WOR sediments, the temperature varies seasonally, not daily, and rain events have minimal impact. Although a 16S rRNA gene from Altiarchaeales has been found in WOR, they do not make visible biomass and are therefore likely less dominant relative to total biomass in estuarine sediments than Altiarchaeales are in German sulfidic springs (Rudolph et al., 2004; Kubo et al., 2012; Probst et al., 2014, 2016).

**FIGURE 2:**
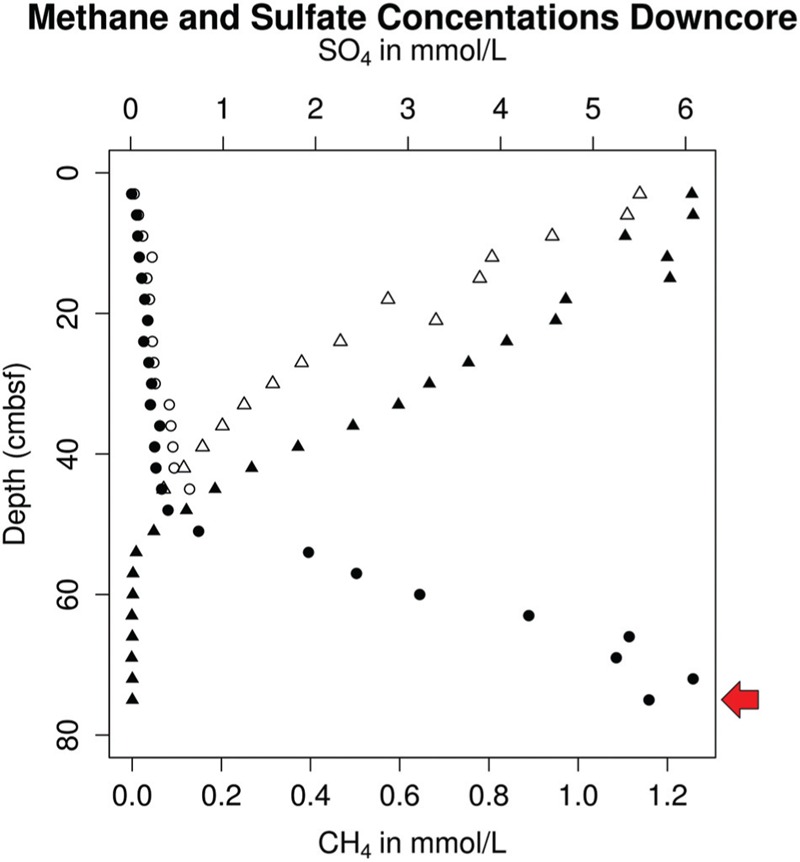
**Aqueous porewater concentrations of sulfate (triangles) and methane (circles) in replicate sediment cores (open/filled-in) taken from White Oak river estuary Station H in October 2012, with a red arrow indicating the depth at which samples for single-cell sorting were taken**.

We obtained a single cell genome from a methane-rich depth (**Figure 2**) in the WOR and used it to recover two genomes from metagenomes from the same site sampled on a different date. Dozens of other novel archaea and bacteria genomes have been derived from this dataset and are described elsewhere (Baker et al., 2015; Lazar et al., 2015; Seitz et al., 2016). By placing these new genomic reconstructions in the context of those previously recovered from sulfidic springs, we investigated the phylogeny of the Altiarchaeales and gained clues about how members of the Altiarchaeales show genetic adaptations for different environments.

## Materials and Methods

### Sampling

Plunger cores (1 m) of sediment were manually retrieved from 1.5 m of water in the WOR Station H (34° 44.490′ N, 77° 07.44′ W) in October 2010 and October 2012. Subsampling of the 2010 sample, and subsequent metagenomic sequencing were described previously (Baker et al., 2015). The 2012 core was sectioned within 30 h of retrieval into 3 cm intervals and samples were removed for methane and sulfate analyses, which were measured with the methods described in Lloyd et al. (2011). Sediments from the 72–75 cm section were used to fill up a 15 ml plastic tube and placed on water ice for transport to the University of Tennessee in Knoxville, Knoxville, TN, USA. After 48 h, 5 ml of anoxic artificial seawater (ASW) and ∼1 g of sediment were added to a 10 ml glass serum vial under nitrogen gas. The serum vial was incubated between 20 and 27°C while shaking at 100 rpm for 48 h.

### Cell Extraction

Cell extraction methods followed a previously published one (Lloyd et al., 2013), with the following minor modifications. The glass serum vial was shaken, the cap removed, and 5 ml of the colloidal mixture were transferred to a sterile 15 ml plastic tube before starting the cell extraction procedure. An additional 5 ml of anoxic ASW was added to the vial and transferred to the 15 ml tube to ensure complete sediment transfer. The tip of a Misonix Microson^TM^ Ultrasonic Cell Disruptor was placed in an ice bath next to the plastic tube containing the sample and manually sonicated two times/s at 20% power to dislodge cells from their associated minerals. Next, the tube was vortexed for 10 s and the sediment was allowed to settle for 10 min. Seven hundred and fifty microliters aliquots of the supernatant were gently laid on top of an equal volume of 60% Nycodenz solution in a sterile 2 ml microcentrifuge tube. The 2 ml tubes were then centrifuged at 11,617 × *g* and 4°C for 1 h. The layer above the Nycodenz gradient was carefully removed and pooled into a sterile 15 ml plastic tube. Lastly, 1.5 ml aliquots were mixed with 375 μl of a 6% betaine 1x TE solution and immediately placed at -80°C.

### Cell Sorting, Amplification, Screening, and Sequencing

A 1:1 mixture of extracted cells and sterile, 0.2 μm filtered, UV treated phosphate buffered saline (PBS) was incubated with 2 μl of 0.5 μM Syto 9 and 0.5 μM Syto 62 dye (Invitrogen) for 1 h in the dark. Two 96 well plates were treated with UV for 30 min before 3 μl of TE were added to each well. The stained cells were gravity filtered through a 30 μm mesh and sorted with a BD Cytopeia Influx Flow Cytometer (Oak Ridge National Laboratory in Oak Ridge, TN, USA) in a class 1000 clean room. One cell was placed in each well based on sidescatter and 640 nm red fluorescence intensity. Sorted cells were lysed and DNA was amplified by multiple displacement amplification (MDA) as described previously (Beall et al., 2014). 16S rRNA genes were amplified from each well after a 1:150 dilution of the MDA products (SAGs) using primers A344f and A915r for Archaea (Stahl and Amann, 1991) and BAC-8F and BAC-1492R for bacteria (Teske et al., 2002). Products were visualized on an agarose gel. Six SAGs amplified with bacterial primers, six with archaeal. Samples amplifying with bacteria or both primer sets were not analyzed further. Samples that were positive for only archaea were confirmed by repeating the amplification. Only one SAG had replicable amplification when archaeal primers were used. After Sanger sequencing (UT Genomic Core) confirmed the SAG was archaeal, it was targeted for whole genome sequencing.

The SAG DNA was quantified by UV absorbance, yielding 14 μg. DNA sequencing was performed on a TruSeq v2 type library with the Illumina MiSeq platform, 250 bp pair-end at the Hudson Alpha Genomics Center. The sample was treated with S1 nuclease prior to library preparation in order to cleave branched DNA structure formed by the MDA process (Zhang et al., 2006).

### SAG Assembly and Annotation

The 19,275,108 sequences totaling over 48 million bp were quality filtered using the CLC Genomics Workbench 6.5.2^1^ using the following criteria: >30 Phred score or trimmed on the ends until that criteria is met. Reads mapping at >90% length and >98% sequence identity to common contaminates [i.e., *Homo sapiens, Escherichia coli* K12 (GCA_000019425.1), *Saccharomyces cerevisiae* (GCA_000146045.2), Phi179 and Phi29] were removed along with duplicated sequences. 17,025,806 paired and 879,071 unpaired sequences between 20 and 251 bp remained. The remaining high quality paired and single reads were assembled using the SPAdes 3.0 assembler with the following parameters: spades.py -m 62 -o <OUTPUT_DIR> –sc –12 <PAIRED_READS> -s <SINGLES> -t 15 -k 21,33,55,77,99,127 –careful. Gene features were identified using Prodigal (2.7) (Hyatt et al., 2010). Gene features were then annotated using Prokka 1.10 using the options: -evalue 0.00001 –addgenes –kingdom Archaea –rfam -prefix<PREFIX> –cpus 4 –outdir <OUTDIR> <CONTIGS> (Seemann, 2014). Ribosomal RNA genes were annotated using barrnap 0.5^2^. To check for contamination we mapped high-quality raw reads to the Greengenes database (DeSantis et al., 2006) and found that all reads mapping to 16S rRNA gene fell within the Euryarchaeota. The dynamic genome assessment tool CheckM was used to assess contamination on the basis of the conserved single copy protein domains (Parks et al., 2015). Duplicate single copy genes identified by CheckM were also checked manually.

### Metagenome Binning

Illumina (HiSeq) shotgun genomic reads were screened against Illumina artifacts (adapters, DNA spike-ins) with a sliding window with a kmer size of 28 and a step size of 1. Reads with three or more N’s or with average quality score of less than Q20 and a length <50 bps were removed. Screened reads were trimmed from both ends using a minimum quality cutoff of 5 using Sickle^3^. Trimmed, screened, paired-end Illumina reads were assembled using IDBA-UD (Peng et al., 2012) with the following parameters: --pre_correction --mink 55 --maxk 95 --step 10 --seed_kmer 55. To maximize assembly reads from different sites were co-assembled, as detailed in Baker et al. (2015, 2016) and Lazar et al. (2015). Initial binning of the assembled fragments was done using tetra-nucleotide frequencies signatures and emergent self organizing mapping (ESOM) as detailed in Dick et al. (2009) and binning was enhanced by incorporating coverage signatures for the assembled contigs (Sharon et al., 2013). Binned contigs associated with the WOR_SM1_SCG were first identified via BLASTN alignments (Altschul et al., 1990). The completeness of the genomes within bins was then estimated using CheckM (Parks et al., 2015). Coverage was determined by recruiting reads to scaffolds by BLASTN (bitscore > 75). Binning was also manually curated based on GC content, top blast hits, and mate-pairings.

### Phylogenetics

Phylogenetic inference followed the methods of Lloyd et al. (2013). The position based gene homology search tool hmmer v3.1b2, Hidden Markov models based on Clusters of Orthologous Genes (COGs), and in-house scripts were combined to locate the 43 conserved genes within 114 archaeal genomes from every archaeal genus having genomic information available on IMG^4^ and assessed to be greater than 50% complete by CheckM (Eddy, 2011; Markowitz et al., 2014; Parks et al., 2015; Huerta-Cepas et al., 2016). The alignment and tree building software ARB was used for trimming and to construct maximum likehood trees, while bootstrap support for 16S rRNA gene trees were assessed using the RaxML rapid bootstrapping method. Additionally, Phylobayes was used to inference concatenated single copy gene trees using eight chains of “pb -d <trimmed_alignment.phy> -cat -gtr -dgam 4 chain<X>”. Chains were ended when the maxdiff was observed to be less than 0.3 using bpcomp -x 500 2 chain1…chain8. ArginyL-tRNA synthetase and nine conserved ribosomal genes (RplS7/L1/L3/L4/L2/L14/L16/S4E/L15) were used in this analysis. A subset of three of these genes present in candidate division pMC2A384 archaeon sp. SCGC AAA252-I15 was used to add the SAG to the tree via maximum parsimony. Sequences were aligned with mafft and trimmed using ARB as detailed previously (Westram et al., 2011; Lloyd et al., 2013). A larger set of 26 conserved marker genes were also considered separately for genomes that contained them; alignments and trees were constructed using default settings in ETE2 (Huerta-Cepas et al., 2010). *In silico* primer alignments and annotation of functional gene alignments were done using the CLC Genomic Workbench 6.5.2^5^.

### Comparative Genomic Analysis

After gene calling and annotation a variety of bioinformatic toolsets were used to compare the seven genomes in the study (**Table 1**). Comparative genomics analysis was performed using methods detailed at http://merenlab.org/2015/11/14/pangenomics/ using Anvi’o platform to visualize the output of ITEP 1.1 (Benedict et al., 2014; Eren et al., 2015). Potential homologs across the genomes were discovered using the default BLAST alignment parameters within ITEP. Proteins clusters were assigned by the Markov Cluster Algorithm (MCL) using the maxbit scoring method with the inflation value set to 2.0 the score cutoff set to 0.4. Homologous gene sets for different groups of Altiarchaeales were determined based on similarity of genes within each subset of genomes, not the presence of a gene with a particular annotation. So, some genes with similar annotations can be present in multiple core sets, although they are different enough from each other that they are not considered homologous.

**Table 1:** Statistics on genomes highlighted in this study.

	Freshwater Altiarchaeales			Estuarine Altiarchaeales		
	MSI_SM1	CG_SM1	IMC4_SM1	WOR_SM1_SCG	WOR_SM1_86-2	WOR_SM1_79
Status	Draft	Draft	Draft	Draft	Draft	Draft
Contigs	467	220	170	300	170	332
Basepairs (Mb)	3.33	1.46	1.37	2.55	2.09	3.19
GC content (%)	32.09	32.73	48.48	37.12	40.83	39.76
N50	7996	8841	9252	16971	15808	10683
Predicted proteins	3005	1388	1478	2514	2297	3275
tRNA	70	37	18	23	23	24
Completeness^a^	95	90	79	81	77	58
Reference	Probst et al., 2014	Probst et al., 2014	Probst et al., 2014	This Study	This Study	This Study

*^a^Calculated by CheckM.*

### Hamus Protein Homolog Comparison

One protein (EMBL accession no. A0A098E857) found to be expressed in *Candidatus* Altiarchaeum hamiconexum (Probst et al., 2014; Perras et al., 2015) was used as a query in a BLAST alignment to all predicted proteins from the six genomes listed in **Table 1** as well as candidate division pMC2A384 archaeon sp. SCGC AAA252-I15 (pMC2A384) using default settings. Each alignment with *e*-value lower than 1 × 10^-60^ was considered as a possible homolog, because we found this value to adequately separate paralogs with similar domains to the hami proteins within the MSI genome. Similarly, BLAST alignments to the putative S_Layer_N domain region (aa position 5–81) in A0A098E857 and hidden Markov model based alignments to Euryarchaeotal S Layer domain proteins (ENOG410KVWW) were also conducted (Altschul et al., 1990; Huerta-Cepas et al., 2016). Potentially homologous alignments were inspected manually in order to check for erroneous alignments to repeat regions and apparent pseudogenes. Then each putative protein was aligned against the UniProtKB database at http://www.ebi.ac.uk/Tools/hmmer/ in order to visualize the alignment to conserved domain architectures.

### Data Archiving

Short reads and assembly from WOR_SM1_SCG were submitted to NCBI BioProject PRJNA321288 (Accession Numbers: SRR3575064 and MCBE00000000). WOR_SM1_79 (Accession Number: MCBD00000000) and WOR_SM1_86-2 (Accession Number: MCBC00000000) were added to the NCBI BioProject PRJNA270657. IMC4_SM1 genome assembly was added to the NCBI BioProject PRJEB6121 (Accession Number: MCBF00000000). The MSI_SM1 genome assembly was previously publically available (Accession Number: CCXY00000000.1). CG_SM1 was previously available in the NCBI short read archive Acc. No. SRR1534154. Geochemical data are housed at www.bco-dmo.org.

## Results

### Geochemical Setting for Altiarchaeales

Sulfate diffusing across the sediment-water interface (6 mM) was mostly depleted by 61.5 cm into the sediments (**Figure 2**). Below this point, methane concentrations increased with depth in a concave up fashion suggesting anaerobic methane oxidation (Martens and Berner, 1977). Methane concentrations reached saturation (∼1.2 mM) near the bottom of the core (70.5 cm; **Figure 2**). A sulfide smell was detected below about 9 cm, consistent with previous cores from this site. The good vertical alignment of the geochemistry in the two cores suggests that this geochemical setting was spatially stable, a result that has been noted previously (Lloyd et al., 2011).

In the WOR metagenomes, 16S rRNA genes for Altiarchaeales were found only at or below the sediment layer where downwardly diffusing SO_4_^2-^ meets upwardly diffusing CH_4_ (SMTZ). 16S rRNA gene sequences that were 100% identical over >900 bp to that of WOR_SM1_SCG were present in the SMTZ (16-24 cm) and methanogenic zone (52–54 cm), and absent in the sulfate-rich zone (8–12 cm). The presence of Altiarchaeales only at or below the SMTZ agrees with previous findings, which only recovered similar 16S rRNA gene sequences from the methanogenic zone (Kubo et al., 2012).

### Genome Quality Assessment

WOR_SM1_SCG assembled to a total size of 2.55 Mbp in 300 contigs above 1000 bp, the largest of which was 62,700 bp (**Table 1**). Contigs from WOR_SM1_79 and WOR_SM1_86-2 showed significant similarity to the single cell genome assembly (Altschul et al., 1990). WOR_SM1_86-2 was from within the SMTZ and WOR_SM1_79 was from the methane-rich zone. WOR_SM1_79 and WOR_SM1_86-2 contained 361 and 170 contigs, totaling 3.19 and 2.09 Mb with maximum contig sizes of 93,451 and 80,876 bp, respectively.

WOR_SM1_SCG appeared to contain only one genome with a single copy each of 16S, 23S, and 5S rRNA gene sequences and a predicted ∼5% contamination in CheckM. Only five putatively single copy conserved genes were duplicated, and two of them shared a contig. Another pair matched each other 100% in the overlapping region, suggesting a sequence assembly error, while a fourth pair appeared to be the result of a mis-annotation of PFAM marker PF01287. The final pair was in PF03950, tRNA synthetase Class I, which was duplicated in 189 out of the 4,227 bacterial and archaeal finished genomes on IMG suggesting these marker genes are naturally duplicated in a small subset of known genomes. After removing 29 contigs containing duplicate gene markers that were suspected contaminants from WOR_SM1_79, and nothing from WOR_SM1_86_2, both genomes from metagenomes were below the suggested 5% contamination cutoff (Parks et al., 2015). The 43 single copy conserved genes used for phylogeny were also used to further assess contamination and visualized using Anvi’o (**Supplementary Figure S1**) (Eren et al., 2015). Some single copy genes appeared in multiple copies due to apparent fission events (discussed below), while other incidents of multiple copies likely indicate the presence of multiple strains within the assemblies. Multiple copies of some genes involved in the replication of DNA also point strain-level contamination (**Data Sheet S1**). The SAG assembled slightly more completely than the genomes from metagenomes, based on the presence of single copy conserved genes (**Table 1**) (Lloyd et al., 2013). The completeness of the three genomes compared favorably with the highest values obtained in similar studies (Wrighton et al., 2012; Lloyd et al., 2013; Rinke et al., 2013; Swan et al., 2014).

### Phylogeny

**FIGURE 3:**
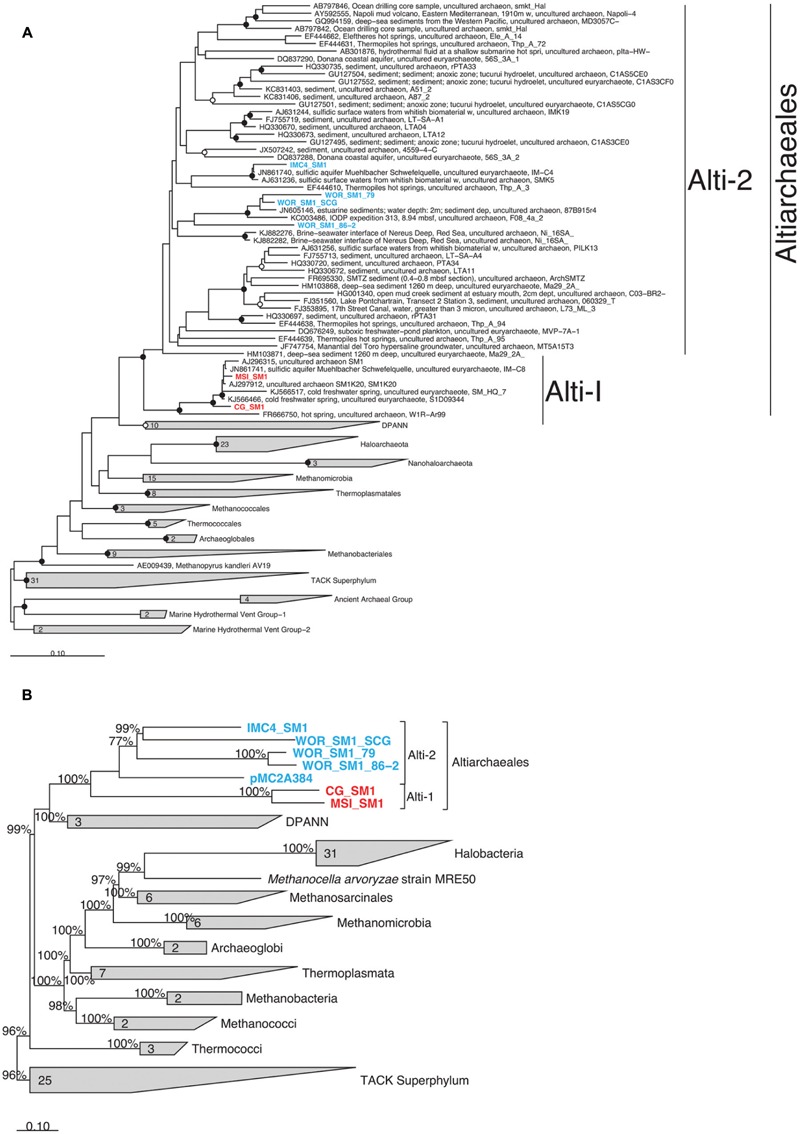
**Phylogenetic placement of Altiarchaeales based on **(A)** a maximum likelihood tree constructed from full length (>1300 bp) 16S rRNA gene sequences; smaller sequences (>900) within the Altiarchaeales group were added to the tree using maximum parsimony (ARB); filled and open circles indicate bootstrap support at greater than 90 and 70%, respectively, **(B)** a Phylobayes tree constructed with 10 conserved universal proteins from 94 archaeal genomes.** The 2220 amino acid positions which were conserved at 30% across the alignment were compared. pMC2A384 was added via parsimony (ARB). Percentages at nodes indicate proportions of trees which agreed with the displayed branching pattern. Sequences were derived from terrestrial springs (red) and estuarine/lacustrine (blue) samples. Scale bars show 10% difference. Gray trapezoids indicate collapsed clades with the number of individuals indicated.

16S rRNA genes were present in six genomes and were monophyletic in the candidate order Altiarchaeales (**Figure 3A**), branching deeply within the Euryarchaeota. The Altiarchaeales branch with the novel phylum DPANN (Rinke et al., 2013), although the low bootstrap support, and variable placement with different phylogenetic tests suggests long-branch attraction. Using a concatenated subset of 10 conserved genes present in the six genomes under consideration, these genomes were monophyletic outside the majority of the Euryarchaeota by Bayesian and maximum likelihood analyses (**Figure 3B**). Candidate division pMC2A384 archaeon sp. SCGC AAA252-I15 was derived from Sakinaw Lake (Rinke et al., 2013), and was not given a taxonomic classification in its initial publication, since it lacked a 16S rRNA gene. We found it to group with the Altiarchaeales according to the three single copy conserved genes that overlapped with the 10 used in this study (**Figure 3B**) and 23S rRNA gene sequences (data not shown). Concatenated gene phylogenies agree with 16S rRNA gene phylogenies that Altiarchaeales is a monophyletic clade branching deeply within the Euryarchaeota, and are likewise susceptible to long-branch attraction with the DPANN.

**FIGURE 4:**
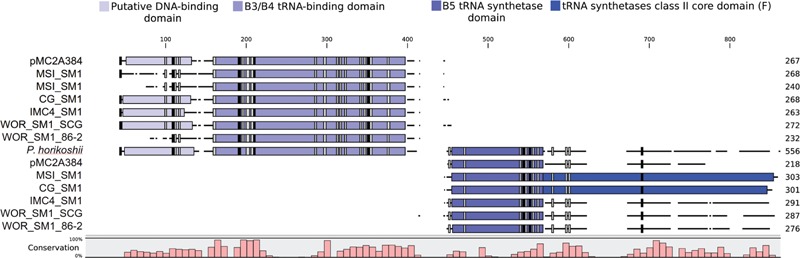
**The amino acids of phenylalanine tRNA-synthetase beta subunit (*pheT*) in six proposed Altiarchaeales genomes, were aligned using ClustalO to 104 *pheT* sequences from one representative of each archaeal genus with draft or finished genomes above 50% completeness, but only *pheT* from *Pyrococcus horikoshii* is shown.** Each aligned gene is labeled by genome name, and the length scale is in amino acids. Colored boxes placed over the aligned sequences represent protein domains associated with *pheT* (InterProScan5). Black and gray boxes denote the presence of conserved residues found in 100 or 90% of archaeal *pheT*, respectively. The histogram tracks the relative conservation of residues across all aligned archaeal *pheT*.

Further evidence for the monophyly of Altiarchaeales comes from a putative phenylalanine tRNA synthetase beta subunit (*pheT*) that occurred in five of the six genomes in **Table 1** and candidate division pMC2A384 archaeon sp. SCGC AAA252-I15. In each genome, it was split just before the B5 domain, which is a putative DNA-binding domain. Alignments of the split *pheT* to representatives from Archaea revealed these genes share nearly all well conserved residues (**Figure 4**). Each half of the split *pheT* gene occurred in the middle of assembled contigs, so the split did not appear to be an artifact from the fragmented genome or an assembly error. Loci where the split genes occur were well supported by reads mapped to these contigs and the split gene was present across the different assemblers used for these genomes (IDBA_UD, SPAdes, and Mira). However, we could not determine their relative placements in the genomes since, in each genome, the two pieces occurred on different contigs. The position near the B5 domain within *pheT*, where the split occurs, shows poor homology across all three domains of life. *Homo sapiens, Saccharomyces cerevisiae, Helicobacter pylori*, as well as several of the aligned archaeal *pheT* genes possessed multiple amino acid insertions in the same location (**Figure 4**) (Safro et al., 2004), however, no currently sequenced genome has a split in *pheT* like we observed in our Altiarchaeales genomes. Additionally, one other split gene was found in all six Altiarchaeales genomes which was annotated as 2-(3-amino-3-carboxypropyl)histidine synthase. This gene appears to be homologous to and share a similar split site with another split gene found in a Nanoarchaeota SAG collected from Obsidian Pool (**Supplementary Figure S2**) (Podar et al., 2013).

Within the Altiarchaeales, the seven genomes showed clear and consistent subdivisions. By 16S rRNA gene sequences, WOR_SM1_SCG, both WOR genomes from metagenomes, and IMC4_SM1 fell into a separate clade, designated Alti-2, from CG_SM1 (USA, UT) and MSI_SM1 termed Alti-1 (**Figure 3A**). Alti-2 contained the majority (88%) of 16S rRNA gene sequences from environmental libraries, including all other sequences from marine sediment. Additionally, one of the sequences in this clade was previously obtained from the same station in WOR sediments (JN605146; Kubo et al., 2012). This clade, however, was not marine/estuarine specific because it also contained 16S rRNA gene sequences derived from cold springs, hot springs, a hydroelectric dam, coastal and hypersaline aquifer, deep sea brines, and lacustrine sediments. In contrast, the clade containing the previously described genomes from CG_SM1 and MSI_SM1 contained only other sequences from cold and hot springs. Mismatches to commonly used 16S rRNA gene primers were found in all Altiarchaeales 16S rRNA gene sequences in this study, so it is likely that environmental abundances of Altiarchaeales have been masked in previous studies (**Supplementary Figure S2**) (Takai and Horikoshi, 2000; Teske et al., 2002; Yu et al., 2005). Sequences from Alti-1 had 1 mismatch to the ARC-8F (Teske et al., 2002), while sequences from Alti-2 had 0 mismatches. Both Alti-1 and Alti-2 have an insertion within the sequence complementary to ARC-1492R (Teske et al., 2002). pMC2A384 appeared to cluster with the other Alti-2 genomes, however, no 16S rRNA gene and only a subset of amino acid positions were available for the phylogenetic analyses used to make this inference.

Within the Alti-2 clade, the 16S rRNA gene sequences from the WOR grouped together, separately from most other sequences, including that of IMC4_SM1. WOR_SM1_SCG had high 16S rRNA gene sequence identity with the other sequence found previously in WOR sediments, JN605146, and WOR_SM1_79 (95.6 and 95.3%, respectively). WOR_SM1_86-2 shared only 83.1% 16S rRNA gene sequence identity to WOR_SM1_SCG, with no closer sequences available from the NCBI database. WOR_SM1_SCG and WOR_SM1_79 rRNA gene sequences clustered with other sequences from marine sediments and IMC4_SM1 clustered with other sequences from sulfidic springs (**Figure 3A**).

Concatenated protein-coding gene trees also reflect the Alti-1 and Alti-2 clade separations suggested by the 16S rRNA gene trees (**Figure 3B**). Branch lengths between IMC4_SM1 and WOR_SM1_SCG were shorter than branch lengths to any other WOR genome in the concatenated protein tree, while in the opposite is true for the 16S rRNA gene tree (**Figure 3**). Out of the 43 conserved genes that were used as phylogenetic marker genes in this study 26 were found to co-occur in WOR_SM1_SCG, IMC4_SM1, and at least one of the two other WOR genomes. In 18 of the 26 protein trees WOR_SM1_SCG and IMC1_SM1 shared the shortest branch lengths among the Alti-2 protein sequences (data not shown). Additionally, the WOR_SM1_79 and WOR_SM1_86-2 appear to be more closely related to each other than any of the other Altiarchaeales. This was consistent with a 99.8% average amino acid identity between WOR_SM1_79 and WOR_SM1_86-2, which indicates they are likely from the same species (Rodriguez-R and Konstantinidis, 2014).

### Comparative Genomics of the Altiarchaeales

**FIGURE 5:**
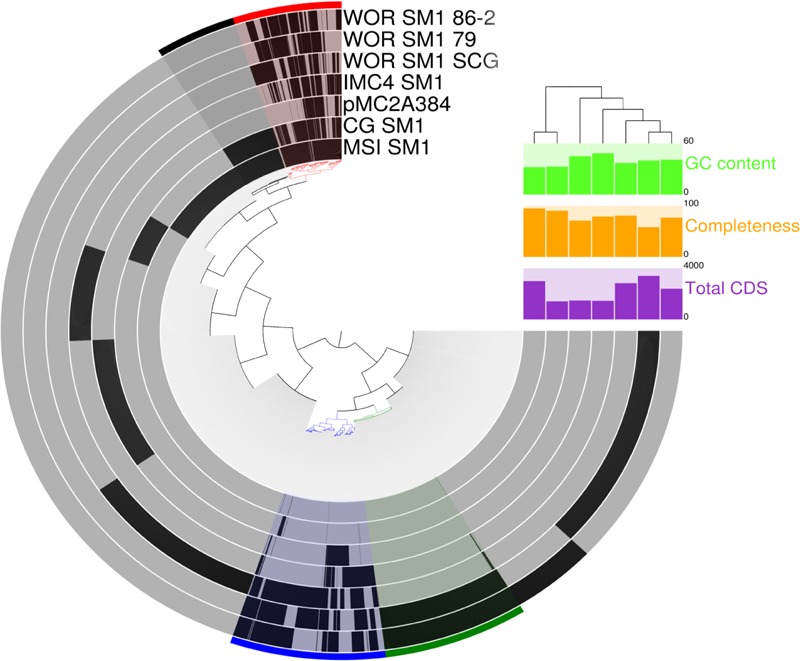
**Comparative analysis of protein clusters within all known Altiarchaeales genomes.** The inner tree was constructed from matrix of protein abundances across the distantly related genomes in this study. Black bars at the tips of inner tree represent the presence or absence of a protein within a genome. Colored bins across the edge highlight co-occurrence patterns across the genomes: core Altiarchaeales proteins (red), core Alti-1 (black), core Alti-2 (blue), and homologous proteins between WOR genomes from metagenomes (green). GC content (bright green), predicted completeness (orange), and total predicted coding sequences (purple) are displayed to the right. Branch lengths in the tree above the histograms correspond to dissimilarities between predicted coding sequences in the genomes.

Only 57 genes were homologous across all seven genomes in this study (**Figure 5**). However, 573 genes were shared between at least some members of the distantly related Alti-1 and Alti-2 genomes and are referred to here after as the “core Altiarchaeales” genes. Although many of these are conserved universally, some of them may be more specific to Altiarchaeales. These include genes associated with oxidative stress, glycolysis, the TCA cycle, transcription/translation, transporters, ATP synthase, pyruvate synthase, acetyl-CoA synthase, F_420_ dehydrogenase, ferredoxin and biosynthesis of amino acids, sugars, lipids, and cobalamins. Also included in the core Altiarchaeales genome are key genes in the Wood–Ljungdahl pathway for CO_2_ fixation such as 5,10-methylenetetrahydrofolate reductase, Acetyl-CoA decarbonylase/synthase complex and carbonic anhydrase, and NADP-dependent methylenetetrahydromethanopterin dehydrogenase. All these Wood–Ljungdahl pathway genes are most similar to genes found in archaea, except NADP-dependent methylenetetrahydromethanopterin dehydrogenase, which has its closest relatives in bacteria. The core Altiarchaeales genome also contains 69 hypothetical proteins.

The core Alti-1 genome, represented by the spring-associated MSI_SM1 and CG_SM1, has 436 shared genes besides those in all the Altiarchaeales genomes. These included glycosyltransferase, lipoprotein *nlpI*, folate synthesis genes, four CRISPR-associated genes, a Type III restriction endonuclease, two Type I restriction endonucleases. Two homologs to glycotransferases, *rfaB*, found only in the Alti_1 set. Putative polysaccharide synthesis genes, which were discussed to be important for biofilm, were exclusive to the Alti-1 set (Probst et al., 2014). Colanic acid export protein, *wza*, was only found in MSI_SM1. 150 hypothetical proteins were also found in the core Alti-1 set (**Data Sheet S2**). The core Alti-2 genome, deduced from five genomes, shared 836 homologous genes including archaeal ATP synthase, cyclic pyranopterin monophosphate synthase, adenylate kinase, flagellar proteins, hydrogenases, and mechanosensitive channels. With the exception of the freshwater member of Alti-2, IMC4_SM1, the shared genes also include sodium specific transporters, calcium binding proteins, sulfite exporter, sulfoxide reductase, adenylylsulfate kinase, and Co-A disulfide reductase.

### Comparison of Putative Hami Proteins

Homologs to the full-length hamus protein from MSI_SM1 were not found in the six other genomes in this study. The hamus protein in MSI_SM1 (EMBL accession no. A0A098E857) is 1808 aa in length and contains three PFAM domains (S_Layer_N, NosD, FKBP_C). A putative homolog, CG_1237, lacked an S_Layer_N domain but contained the FKBP_C domain and was significantly truncated (372 aa vs. 1808). IMC4_884 was also significantly truncated (918 aa vs. 1808), but it was the only putative homolog that contains a putative S_Layer_N domain. Additionally, like the hamus protein found in MSI_SM1, it was predicted to be localized to the membrane with an N terminal signal sequence and a predicted C terminal trans membrane domain (Möller et al., 2001; Perras et al., 2015). A putative homolog, SCG_1643, which contained FKBP_C and Thioredoxin_4 domains, was more similar to another protein in MSI_SM1, a putative peptidylprolyl isomerase (EMBL A0A098E9R9), and was also much shorter at 613 aa. WOR_SM1_79 had another potential homolog, 79_2061, but this was very similar to SCG_1643, with alignments of the two only having 10 amino acid changes with no gaps. 86-2_679 had no similar PFAM domains to A0A098E857, yet it has no other significant matches within the UniProtKB database.

## Discussion

### Phylogeny of the Altiarchaeales

Rudolph et al. (2001) first reported a conspicuous cold-loving biofilm-forming archaea. They named SM1 after the Bavarian spring Sippenauer Moor. Subsequent studies revealed a broad diversity of 16S rRNA gene sequences distantly related to those organisms (Rudolph et al., 2004; Probst and Moissl-Eichinger, 2015), most of which were found in environments with no conspicuous mats (**Figure 1**). By including genomes from estuarine sediments, we identified two major groups within the Altiarchaeales, Alti-1 and Alti-2. Alti-1 is composed primarily of sequences isolated from spring environments, while Alti-2 sequences were from a variety of primarily other types of anoxic environments. We show that only sequences from environments with actively moving water fall into the relatively low-diversity sister clade Alti-1. This clade is distinct from the more widespread, diverse group of Alti-2, which is in agreement with previous 16S rRNA gene surveys (Probst et al., 2014; Probst and Moissl-Eichinger, 2015). These data should be interpreted with caution as mismatches in commonly used 16S rRNA gene primers have likely left the diversity of either clade in a given environment under-sampled. Although IMC4_SM1, a member of the Alti-2, was extracted from the same sample as MSI_SM1, it was shown by qPCR and florescence *in situ* hybridization (FISH) to be in low abundance within the biofilm (Probst et al., 2014). Given the large distance between Alti-1 and Alti-2 (>20% dissimilarity by 16S rRNA genes), we sought to determine whether these two clades are truly monophyletic. Our 16S rRNA gene tree showed good statistical support for the monophyly of the Altiarchaeales sister clades, in agreement with previous work (Probst et al., 2014). However, single copy gene trees from whole genomic constructions have been hindered by lack of representation of genomes from the more widespread Alti-2. The inclusion of our three WOR genomes and one from Sakinaw Lake now supplement the previously known IMC4_SM1 to bring the representation from Alti-2 to five genomes. These additional genomes have good posterior probability support for the conclusion that the monophyletic lineage, Altiarchaeales, is composed of two distinct clades, Alti-1 and Alti-2.

Further support for the monophyly of these clades comes from shared traits encoded by the genomes in this study. The acquisition or loss of genes involved in genome replication have been shown to be helpful in describing the shared evolutionary histories of archaea phyla (Raymann et al., 2014). All Alti-1 and Alti-2 genomes have the same set of replication genes (**Data Sheet S1**) (Probst et al., 2014). In addition to shared replication machinery, synapomorphic mutation were observed in phenylalanine tRNA beta subunit *pheT* in six of the seven genomes and in 2-(3-amino-3-carboxypropyl)histidine synthase in all the genomes. Gene fissions are rare events that can be used to support phylogenies. Shared split genes in Nanoarchaeota have been used to support a relationship between terrestrial and marine hyperthermophilic biotypes despite the genome plasticity that is commonly associated with parasitic lifestyles (Waters et al., 2003; Podar et al., 2013). *pheT* and 2-(3-amino-3-carboxypropyl)histidine synthase are present in a single copy in nearly all other archaea. The fact that they are split in Altiarchaeales suggests that apparent duplications of these genes reported in this and previous work may actually be fission events (**Supplementary Figure S1**) (Probst et al., 2016). Based on this evidence and the placement of candidate division pMC2A384 archaeon sp. SCGC AAA252-I15 within **Figure 3B**, we propose that pMC2A384 belongs within the larger Altiarchaeales classification. Precedent for split tRNA synthase genes occurs in *Aquifex aeolicus*, whose leucine-tRNA synthetase gene is split into two pieces at the tRNA-binding domain site (Ma et al., 2006) and Nanoarchaeota which have split Glu-tRNA_Gln_ amidotransferase and Alanyl-tRNA synthase (Waters et al., 2003; Podar et al., 2013). While a similar split 2-(3-amino-3-carboxypropyl)histidine synthase was observed previously in a single Nanoarchaeota SAG, the lack of this split in other Nanoarchaeota genomes and the lack of phylogenetic evidence linking Nanoarchaeota to Altiarchaeales leads us to concluded that these represent two separate evolutionary events. Protein fission events have been well documented in the literature as a mechanism of new gene formation in prokaryotes (Snel et al., 2000; Suhre and Claverie, 2004; Kummerfeld and Teichmann, 2005; Pasek et al., 2006). The function of this split cannot be determined from the primary sequence, but it is likely to be related to an evolutionary event occurring in an ancestor of these organisms that produced a novel variant of the universally conserved phenylalanine tRNA-synthetase beta subunit. Additionally, the split occurs between two protein domains and nearly all conserved residues are present (**Figure 4**) suggesting that the split *pheT* gene is functional, and recombines post-translationally. This apparent gene-splitting event appears to be a unique character that helps to define this candidate order.

Our results partially agree with placement of Altiarchaeales within the Euryarchaeota (Probst et al., 2014). It should be noted that our inferences include members of the candidate phylum Woesearchaeaota (Castelle et al., 2015) that were not available during earlier analyses (Probst et al., 2014). Our results disagree with previous results showing robust placement of Altiarchaeales (labeled Altiarchaeota in that paper) within the DPANN superphylum in a clade that includes another archaeon with a double-membrane, the ARMAN group (Seitz et al., 2016). It is important to note that while DPANN and Altiarchaeales appear as sister groups in **Figure 3**, we found no statistical support for this branching pattern, suggesting this is a long branch attraction artifact. Finally, each of these results differ from recent work which suggests that Altiarchaeales could be a separate phylum-level lineage distinct from either the DPANN and Euryarchaeota (Hug et al., 2016). Six of the 10 marker genes used in concatenated gene trees in **Figure 3B** overlapped with the 16 marker genes used in two of these previous works (Hug et al., 2016; Seitz et al., 2016). Such wide disagreement between tree topologies warrants further study and suggests Altiarchaeales is quite distantly related to all other taxa studied to date. Our phylogenetic and trait-based analysis suggest that the genomes in this study belong to two distinct, yet monophyletic clades which may belong within a higher taxonomic classification than previously suggested, although for now we conservatively leave them in the Euryarchaeota (Probst et al., 2014).

When comparing the tree topologies between 16S rRNA gene and protein trees within Alti-2, we find some inconsistencies. 16S rRNA gene sequences suggest WOR_SM1_SCG is most closely related to WOR_SM1_79, while protein sequences suggest IMC4_SM1 shares a more recent ancestor with WOR_SM1_SCG. In contrast to the ambiguous position within the Alti-2 for WOR_SM1_86-2 in 16S rRNA gene analyses, protein sequence trees suggest WOR_SM1_86-2 is very closely related to WOR_SM1_79. Data from 16S rRNA genes obtained from metagenomes should be interpreted with caution, as it is often difficult to bin and assemble 16S rRNA gene sequences because of the presence of highly conserved regions and divergent genomic signatures.

### Genome Comparisons within the Altiarchaeales

#### Variation in Genome Size

Across the Altiarchaeales genomes there was considerable variation in GC content and projected genome size. Previous analysis of synonymous vs. non-synonymous mutations within housekeeping genes suggests the MSI_SM1 draft genome represents multiple strains of *Candidatus* Altiarchaeum hamiconexum (Probst et al., 2014). Analysis of housekeeping genes within the other draft genomes presented here also reveals the potential for contamination from multiple strains to a lesser extent. Draft genomes from the WOR are larger than those from terrestrial spring, however, the lack of complete genomes and the considerable evolutionary distance between the genomes impairs our ability to draw conclusions about environmental pressures which may influence genome size and GC content.

#### Genomic Evidence for Hami

Genomes from the Alti-1 and Alti-2 were compared in order to establish functional traits that help to define the group as a whole as well as to identify potential functions useful within their particular environments. Since the grappling-hook-like hami are a defining physical characteristic of *Candidatus* Altiarchaeum hamiconexum, we investigated the other genomes to determine whether this feature is characteristic of all Altiarchaeales. The hamus structure has likely diverged from ancient S-layer proteins following the acquisition of a double membrane structure (Perras et al., 2015). We found evidence for the hamus protein in the Alti-2 clade in IMC4_SM1 genome, however, future work should seek to determine if these proteins are expressed *in situ*. No such homologs containing S_Layer_N domains were observed in CG_SM1 although new evidence suggests CG_SM1 nonetheless have hook-like appendages (A. Klingl, personal communication). None of the genomes from the WOR possessed hami protein homologs with S_Layer_N domains. This points to adaptive utility of hami in the actively flowing stream environment. Given the dramatic differences in length and domain architecture observed in hami protein homologs in this study, future studies should highlight the diversity of form among hami ultrastructures in the Altiarchaeales.

#### Biofilm Production

The Alti-1 core genome contains other genes possibly associated with biofilm formation that are absent in the Alti-2 core genome. The two Alti-1 specific glycosylases have similar domain architecture and homology to *rfaB*, the gene for UDP-galactose:(glucosyl)lipopolysacchride-1,6-D-galactosyltransferase. This gene has been linked to lipopolysaccharide (LPS) synthesis in *E. coli* and mutants appeared to be impaired in UDP-galactose and UDP-glucose catabolism (Qian et al., 2014). UDP-galactose catabolism is also required for *Bacillus subtilis* to survive in a biofilm, but is not required for planktonic growth (Chai et al., 2012). When lipoprotein NlpI, found only in Alti-1 genomes, is disabled in *E. coli*, the cells have a 35-fold decrease in their ability to adhere to intestinal lining (Barnich et al., 2004). This, in combination with the common participation of lipoproteins in biofilm formation (Raaijmakers et al., 2006), suggests that these genes are important for biofilm formation in Alti-1. Homologs of colanic acid export proteins *wza* as well as putative polysaccharide synthesis genes, which were previously discussed to be important for biofilm production (Probst et al., 2014), were only found in MSI_SM1. Mutants for the *wza* enzyme in a *Klebsiella pneumonia* model system have been found to be deficient in producing biofilms (Balestrino et al., 2008; Wu et al., 2011). While Altiarchaeales species are the dominant organism in Crystal Geyser spring water, biofilms similar to those seen in the German sulfidic springs have never been reported there (Probst et al., 2014, 2016). Biofilm formation may be an important trait for some Alti-1 members, while in other environments Alti-1 may use other mechanisms to meet the challenges of living within actively flowing streams.

#### Potential Adaptations and Metabolic Similarities

The core genome of the Alti-2 clade suggested adaptations to a free-living state in sulfidic environments. A few of the genes that differentiate them from Alti-1 are important to energy maintenance, such as ATP synthase, hydrogenases, and adenylate kinase which modulates intracellular ADP:ATP ratios. The presence of flagellar genes suggests that motility may be important in the free-living state. Quite a few Alti-2 genes that suggest adaptations to an estuarine environment, bolstered by the fact that they are absent in IMC4_SM1, the one Alti-2 genome from a freshwater environment. These include osmoregulation mechanisms such as sodium transporters and mechanosensitive channels. In addition, Alti-2 seems capable of sulfur metabolism, since it contains many sulfur-related genes. The presence of these genes could be for sulfur assimilation, but many of them involved in energy-conserving redox reactions. However, no dissimilatory sulfite reductase genes were found. When one of the sulfur-related genes, CoA-disulfide reductase, is knocked out in *Pyrococcus furiosus* growing in the presence of sulfur compounds, the organism loses its ability to survive transient exposure to oxygen (Herwald et al., 2013). Therefore, Altiarchaeales in the WOR may shuttle electrons between sulfur compounds for oxidative stress defense.

Despite all the phylogenetic and functional differences between Alti-1 and Alti-2, there are key similarities, namely the presence of many elements of glycolysis, the tricarboxylic acid cycle, cobalamin production/recycling, and all the genes in the Wood–Ljungdahl pathway for autotrophy, as well as a lack of convincing evidence for catabolism of a broad range of heterotrophic substrates. Alti-1 has previously been suggested to have acquired its NADP-dependent methylenetetrahydromethanopterin dehydrogenase gene from a horizontal gene transfer from a bacterium (Probst et al., 2014). The presence of this gene, also with a potentially bacterial origin, in our Alti-2 genomes supports the possibility that the modified Wood–Ljungdahl pathway seen in *Candidatus* Altiarchaeum hamiconexum is conserved in the Altiarchaeales.

## Conclusion

The three novel genome reconstructions presented here belong to the monophyletic archaeal lineage Altiarchaeales, which can be divided into two clades, Alti-1 and Alti-2. Moreover, these genome reconstructions belong to the more diverse and widespread Alti-2 clade. Our analyses supports previous claims that Altiarchaeales is a deeply branching order within the Euryarchaeota, but further research on deeply branching archaeal lineages is necessary in order to resolve tree topologies with the recent expansion of the DPANN superphylum. The core genes shared by all known genomes have a characteristic split *pheT* gene, and genes for a version of the Wood–Ljungdahl pathway. However, Alti-1 and Alti-2 had genomic features that distinguished them from each other and suggest adaptations to different environments. Alti-1 have adaptations to form hooks in high flow environments and may form biofilms, while the members of Alti-2 inhabiting saline environments have adaptations that make them specialized for the free-living state in a sulfidic, saline environment.

## Author Contributions

JB and KL designed the study. All analyses were completed by JB, KL, MP, and BB. Other genomic data for comparative analyses was provided by BB and AP. All authors contributed to the discussion of the results. Manuscript was written by JB with inputs from KL, BB, MP, and AP.

## Conflict of Interest Statement

The authors declare that the research was conducted in the absence of any commercial or financial relationships that could be construed as a potential conflict of interest.

## Footnotes

1 https://www.qiagenbioinformatics.com/

2 http://www.vicbioinformatics.com/

3 https://github.com/najoshi/

4 "http://img.jgi.doe.gov/

5 https://www.qiagenbioinformatics.com/

## Supplementary Material

FIGURE S1 **Single copy conserved genes in genomes.** Histograms are labeled with the gene name and *y*-axis depict the number of positive hits to hmmer models in a set of 43 single-copy genes which are conserved in most archaea (Lloyd et al., 2013). The 1-D plot summarize the data and the mode is used to estimate the number of genomes in the assembly.

FIGURE S2 **Mismatches in common full length archaeal primers.** Primer sequence alignment and predicted restriction digestion sites are annotated on the full length 16S rRNA gene sequence. Green annotations were assigned by the primer alignment software (CLC Genomics Workbench 6.5.2). Ambiguous base calling prevented the alignment of ARC-1492R to WOR_SM1_79. WORSM1-9f (5′-CCAGTTGATCCTGCTGG-3′) and WORSM1-1405r (5′-CCACTCGATTGGTTTGAC-3′) were designed for this study.

Data Sheet S1 **Homologs of archaeal replication machinery in Altiarchaeales genomes.** An *X* indicates the presence of a gene in the genome.

Data Sheet S2 **Workbook which contains abundances and annotations for each homologous protein identified during the comparative genome analysis.** Each worksheet in this datasheet corresponds to the co-occurrence patterns described in **Figure 5**.
