# Supplementary figures and images for: Culture Independent Genomic Comparisons Reveal Environmental Adaptations for Altiarchaeales
Source: Front Microbiol. 2016 Aug 5;7:1221. doi: 10.3389/fmicb.2016.01221 (PMC4975002; doi:10.3389/fmicb.2016.01221)

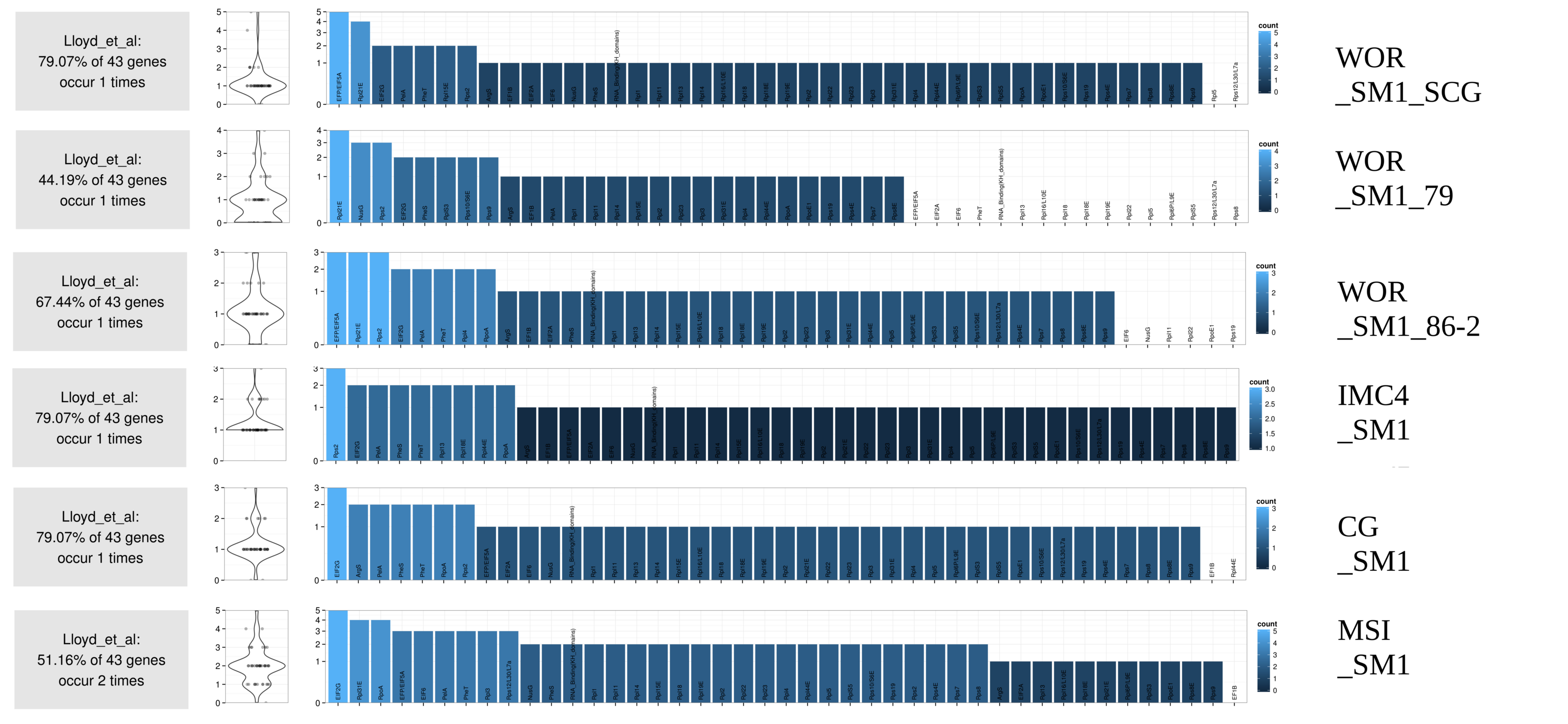

Supplement: FIGURE S1 — Single copy conserved genes in genomes. Histograms are labeled with the gene name and y-axis depict the number of positive hits to hmmer models in a set of 43 single-copy genes which are conserved in most archaea (Lloyd et al., 2013). The 1-D plot summarize the data and the mode is used to estimate the number of genomes in the assembly. [file Image_1.TIFF]

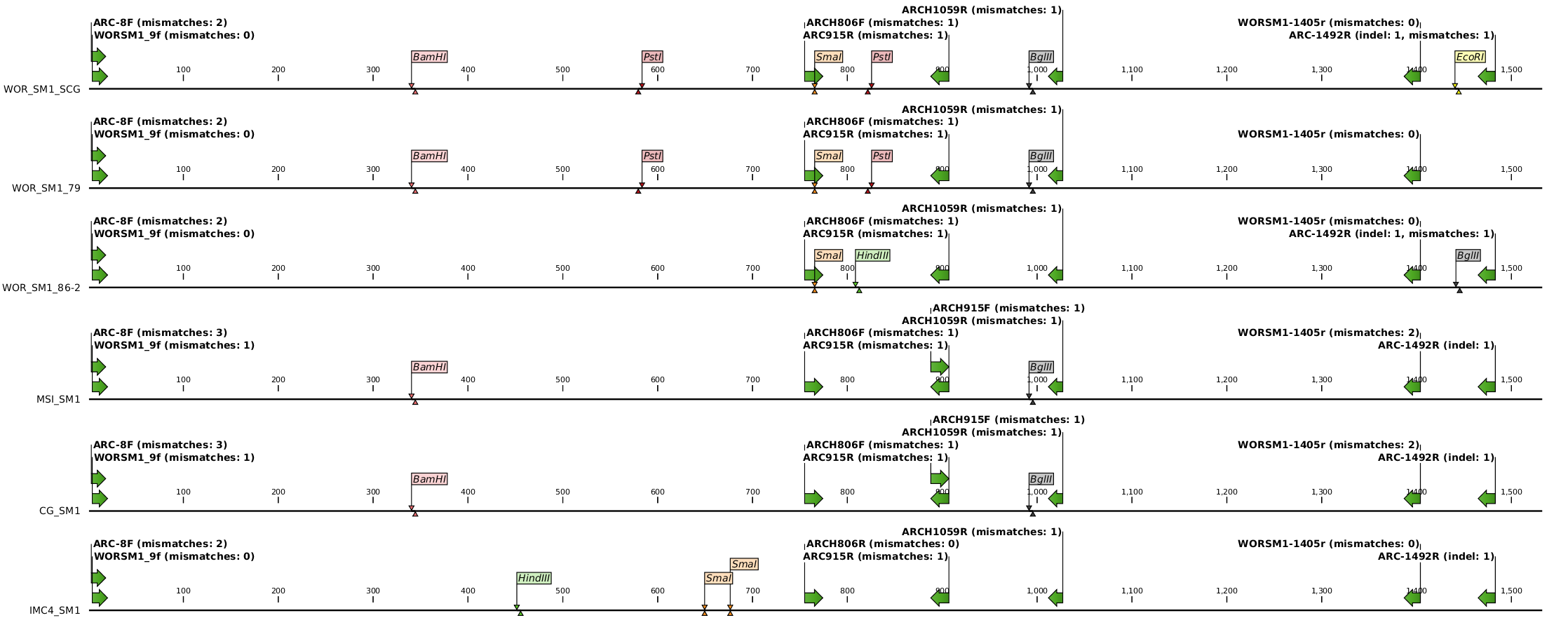

Supplement: FIGURE S2 — Mismatches in common full length archaeal primers. Primer sequence alignment and predicted restriction digestion sites are annotated on the full length 16S rRNA gene sequence. Green annotations were assigned by the primer alignment software (CLC Genomics Workbench 6.5.2). Ambiguous base calling prevented the alignment of ARC-1492R to WOR_SM1_79. WORSM1-9f (5′-CCAGTTGATCCTGCTGG-3′) and WORSM1-1405r (5′-CCACTCGATTGGTTTGAC-3′) were designed for this study. [file Image_2.TIFF]
